# Physical and Psychological Effects of Home-Based vs Face-to-Face Prehabilitation in Individuals With Severe Obesity Awaiting Bariatric and Metabolic Surgery: Protocol for a Randomized Controlled Trial

**DOI:** 10.2196/87053

**Published:** 2026-08-28

**Authors:** Darlan Laurício Matte, Ananda Quaresma Nascimento, Alexandro Andrade, Guilherme Torres Vilarino

**Affiliations:** 1 Center for Health and Sports Sciences, Universidade do Estado de Santa Catarina, Pascoal Simone, 358, Coqueiros, Florianópolis, 88080-350, Brazil, 55 48999239498; 2 Postgraduate Program in Human Movement Sciences (PPGCMH), Center for Health and Sports Sciences, Universidade do Estado de Santa Catarina, Florianópolis, Brazil; 3 Postgraduate Program in Physiotherapy (PPGFT), Center for Health and Sports Sciences, Universidade do Estado de Santa Catarina, Florianópolis, Brazil

**Keywords:** bariatric surgery, BMI, physical exercise, mental health, anxiety, depression

## Abstract

**Background:**

Obesity is a growing public health issue associated with comorbidities and substantial medical costs. Although bariatric and metabolic surgery (BMS) is often recommended for individuals with severe obesity, prehabilitation may optimize their physical and psychological status. However, face-to-face delivery can limit accessibility, and evidence on home-based approaches remains scarce.

**Objective:**

This study protocol aims to compare the effectiveness of a home-based prehabilitation program with a face-to-face prehabilitation program on physical and psychological variables in individuals with severe obesity awaiting BMS.

**Methods:**

This is a study protocol for a randomized, controlled, parallel-group trial. To ensure ecological validity, recruitment will be stratified to target 50% low-income and 20% rural participants. Participants with grade III obesity and an indication for BMS will be allocated via block randomization to 3 groups (1:1:1): a face-to-face group, a home-based group, and a control group. The intervention will last 7 weeks (1 week of familiarization plus 6 weeks of training) and will include 12 combined aerobic and resistance exercise sessions and 3 health education sessions. The primary outcome will be the total score of the Hospital Anxiety and Depression Scale (HADS). Secondary outcomes include the HADS subscales (HADS-A and HADS-D), sleep quality, mood states, self-efficacy, quality of life, and functional performance (6-minute walk test, handgrip strength, and 5-repetition sit-to-stand test).

**Results:**

This study was funded in August 2025 by the Research Support Program of the Santa Catarina State University. Recruitment is scheduled to begin in April 2026. The intervention phase is expected to be completed in September 2026, followed by data analysis between October and November 2026. Results are expected to be published in mid-2027.

**Conclusions:**

This trial will provide evidence on the comparative effectiveness of home-based and face-to-face prehabilitation for individuals with severe obesity. The findings can be used to support the implementation of feasible, accessible, equitable, and cost-effective preoperative care models.

## Introduction

Obesity is a health condition that has grown significantly over the past 50 years [1], affecting approximately 2 billion adults [2]. It is estimated that one-third of the global population is overweight or obese [1], which represents a major public health problem due to its association with multiple diseases [3-5].

Obesity is characterized by the excessive accumulation of adipose tissue and is recognized when the BMI is ≥30 kg/m^2^. Severe obesity is classified when the BMI is ≥40 kg/m^2^ or at least 35 kg/m^2^ with associated comorbidities [6]. Recently, a new classification has been proposed that distinguishes “preclinical obesity,” marked by excess fat without functional impairment, from “clinical obesity,” characterized by organ and tissue damage. This framework suggests associating BMI with additional indicators, such as waist circumference and body composition, for a more precise assessment of health status [7].

Obesity is often associated with excessive calorie consumption and low levels of physical activity (PA), leading to an energy surplus [8,9]. However, genetic and environmental factors are also responsible for weight gain [2]. In addition, obesity is associated with several comorbidities, including diabetes mellitus, dyslipidemia, and hypertension [3]; cardiovascular diseases; obstructive sleep apnea and chronic obstructive pulmonary disease (COPD) [5,10]; cancer [4]; morbidity and mortality from chronic diseases; and premature death [10,11]. Other disorders, such as depression and anxiety, are also linked to obesity, sometimes as contributing factors and, in other cases, as consequences of stigma and discrimination, negatively affecting mental health [8,12].

It is estimated that people with obesity incur 30% higher health care costs than those with a BMI within the range considered healthy [1]. In Brazil, data from the Brazilian Association for the Study of Obesity and Metabolic Syndrome show that people with obesity spend 15% of their income on health care treatments, rising to 30% in severe cases. This economic impact underscores the need for studies that explore effective treatment strategies to support both patients and health care systems.

Although exercise, diet, and psychological support are recommended for the management of obesity, these strategies are complicated for individuals with severe obesity to follow, due to mobility limitations, exercise barriers, and difficulties in preparing meals [13,14]. Thus, in many cases, bariatric and metabolic surgery (BMS) is indicated [15,16].

BMS procedures alter the gastrointestinal tract to decrease calorie intake or absorption [16]. Despite being relatively safe, all surgical procedures carry risks, including complications and adverse outcomes. Postoperative complications occur in approximately 30% of major abdominal surgeries [17,18]. Because obesity is itself a surgical risk factor, patients must be adequately prepared to cope with the physiological stress of surgery. In this context, prehabilitation programs have been proven to be fundamental in improving surgical outcomes [12,15,19].

Despite the importance of prehabilitation, its implementation remains inconsistent, and low adherence reduces program effectiveness. To address this inadequacy, several studies have explored remote prehabilitation strategies [20]. Home-based intervention programs have grown in recent years, especially since the COVID-19 pandemic [20-22], and research has been conducted to test the effects of these programs in populations with fibromyalgia [22], older adults [23], and patients with heart disease [24]. Psychological aspects are important in the prehabilitation process, influencing participation and outcomes. It is also known that physical exercise can promote positive results in variables such as sleep [25,26], mood [27,28], quality of life [29,30], and depression and anxiety [31,32]. However, there is still a lack of prehabilitation studies specifically designed for individuals with obesity awaiting BMS. Comparative trials of home-based vs face-to-face programs are particularly scarce.

Home-based prehabilitation supported by digital technologies can empower patients to take an active role in preparing for surgery and managing their health [33]. Digital health intervention programs can help overcome geographical barriers and enhance access to care for patients, health care professionals, and health care systems. These tools have been evaluated as strategies to deliver surgery-related education, support self-monitoring and goal-setting, provide reliable information, and increase preoperative and postoperative engagement [34].

In BMS prehabilitation, health education is often integrated with exercise, offering individualized follow-up and identification of barriers, teaching of behavioral strategies, goal setting, and guidance in the practical application of exercises [35]. Sessions typically address nutrition, PA, behavioral modification, and obesity-related knowledge, thereby supporting lifestyle change and patient engagement [36].

Despite these advances, direct comparisons between home-based and face-to-face prehabilitation programs that integrate PA measures with mental health outcomes are limited. Therefore, this protocol study aims to compare the effectiveness of a home-based prehabilitation program and a face-to-face prehabilitation program on physical and psychological variables in individuals with severe obesity awaiting BMS. Thus, this study aims to address this gap by evaluating the effectiveness and feasibility of a structured 7-week, exercise-based prehabilitation program for individuals with severe obesity awaiting BMS.

## Methods

### Study Design

This is a study protocol for a randomized, controlled, parallel-group trial, developed in accordance with the SPIRIT (Standard Protocol Items: Recommendations for Interventional Trials) guidelines (Checklist 1) [37]. Table 1 presents the schedule of enrollment, interventions, and assessments.

**Table 1.** SPIRIT (Standard Protocol Items: Recommendations for Interventional Trials) 2025 schedule of enrollment, interventions, and assessments for the randomized controlled trial. Adapted according to the SPIRIT 2025 statement.

	Enrollment	Study period				
		Allocation	Postallocation			
Timepoint	–T1	0	T1 (baseline)	Familiarization	T2 (intervention)	Follow-up
Enrollment						
Eligibility screen	✓					
Informed consent	✓					
Allocation		✓				
Interventions						
Combined exercise face-to-face				✓	✓	
Home-based combined exercise				✓	✓	
Control				✓	✓	
Assessments						
Anthropometric measurements			✓			✓
Sit-to-stand test			✓			✓
Handgrip strength			✓			✓
6-minute walk test			✓			✓
Physical Exercise Self-Efficacy Scale			✓			✓
World Health Organization Quality of Life–BREF			✓			✓
Brunel Mood Scale			✓			✓
Pittsburgh Sleep Quality Index			✓			✓
Hospital Anxiety and Depression Scale			✓			✓

### Ethical Considerations

The study was approved by the Research Ethics Committee of the Santa Catarina State University (UDESC), Brazil (CAAE 88170725.1.0000.0118) and registered in the Brazilian Clinical Trials Registry (ReBEC; registration: RBR-59xgczn). All participants will provide written informed consent. Although blinding of participants and providers is not feasible due to the nature of the interventions, outcome assessments will be performed by independent evaluators blinded to group allocation, with interrater reliability established in pilot assessments conducted in 2025 (intraclass correlation coefficient=0.95). Statistical analyses will be conducted by a blinded statistician, with group codes disclosed only after completion of the primary analyses.

### Participants

Recruitment will occur through dissemination via social media, institutional websites, hospitals, primary health care units, and medical clinics. To mitigate selection biases and promote equity in access to research, the sampling design provides stratified recruitment: 50% of the sample will consist of low-income individuals, and 20% will be residents of rural areas. Verification of these demographic characteristics will occur during the initial telephone contact, at which time the objectives, risks, and benefits of the study will also be presented to potential participants. This will be followed by an initial interview to obtain informed consent and initiate data collection. Data collection will take place at the UDESC, in the Health and Sports Science Center.

**Figure 1.**
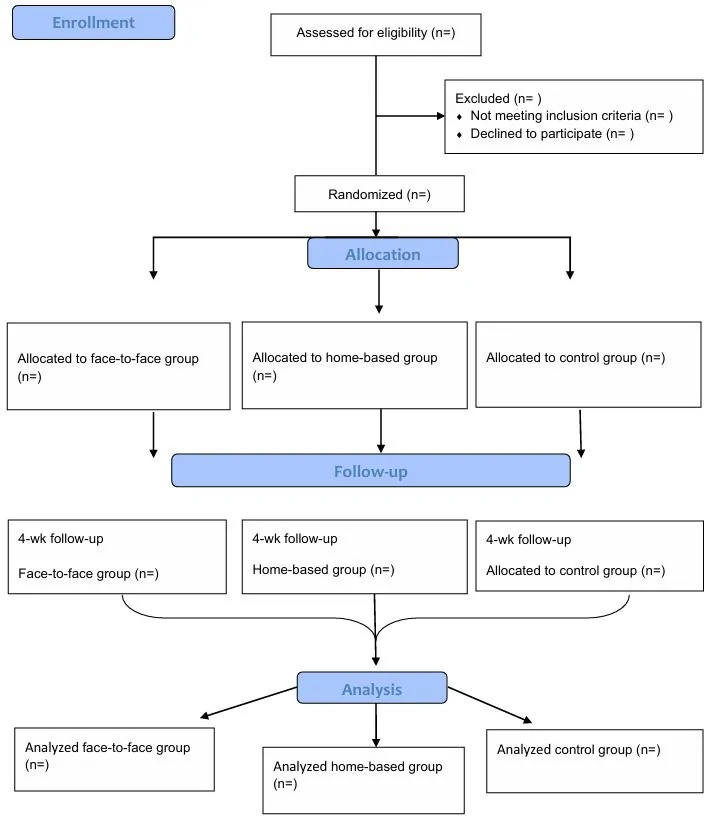
Flow diagram of the study participants according to the CONSORT (Consolidated Standards of Reporting Trials).

Participants will be randomized to 1 of 3 groups: a face-to-face exercise group (FxF), a home-based exercise group (HBG), or a control group (CG) (Figure 1).

### Eligibility Criteria

Individuals of both genders with grade III (severe) obesity who meet the following criteria will be included in the research: (1) BMI>40 kg/m^2^, (2) indication for BMS, (3) formal indication for preoperative physical therapy signed by the BMS surgeon, in accordance with the multidisciplinary guidelines for BMS, (4) absence of comorbidities that compromise safety or the ability to perform the exercises and functional tests, and (5) no participation in any structured exercise program in the past 3 months. To ensure equitable access and diversity, recruitment will be stratified to achieve a sample composition of 50% low-income individuals (defined as monthly household income per capita ≤0.5 minimum wage or total household income ≤3 minimum wages) and 20% residents of rural areas. In addition, participants will be excluded if they meet any of the following criteria: (1) presence of a pacemaker, history of myocardial infarction within the previous 3 months, or unstable angina pectoris; (2) sustained or episodic cardiac arrhythmias aggravated by PA, symptomatic peripheral vascular disease, or any clinical condition representing a risk (eg, inability to safely tolerate heart rate increases >20 beats/min during exercise); (3) clinical diagnosis of acute or chronic respiratory diseases that compromise the safety of physical tests (eg, COPD and asthma with remodeling) or peripheral oxygen saturation (SpO₂) <92% during the 6-minute walk test (6MWT); (4) history of previous BMS or abdominal or thoracic surgery within the previous year; (5) abusive use of or chemical dependence on psychoactive substances (including alcohol and illicit drugs), according to clinical criteria or previous diagnosis; (6) cognitive or physical changes that prevent performance of the tests; and (7) initiation of another form of physical training during the intervention period.

### Randomization and Blinding

After the initial assessment, participants will be allocated through block randomization to receive different interventions performed by physical education professionals and physiotherapists. Participants will be randomly assigned in a 1:1:1 ratio to the HBG, FxF, or CG groups. The randomization sequence will be computer-generated using Sealed Envelope [38], with fixed block sizes of 6 to ensure balanced group allocation throughout recruitment.

Allocation concealment will be ensured using sequentially numbered, opaque, sealed envelopes prepared by an independent researcher who is not involved in recruitment, assessment, or intervention delivery. Envelopes will be opened only after the completion of baseline assessments, minimizing the risk of allocation prediction and selection bias.

The researcher responsible for recruitment, eligibility, and evaluation, as well as statistical analysis, will be unaware of the group to which the individual belongs (blinding of the evaluator and statistician). Due to the type of research, it will not be possible to blind the physical education professionals and physiotherapists responsible for the intervention. Stratification applies only to the recruitment process and not to the randomization procedure.

### Interventions

#### Intervention Program

Participants will complete 12 training sessions (twice weekly) spread over 7 weeks. The first week will be devoted to familiarization with the exercise protocol, while the subsequent 6 weeks will correspond to the actual intervention phase. Assessments will be conducted at baseline, immediately after the intervention is completed, and at a 4-week follow-up. The 4-week follow-up is included to evaluate the short-term maintenance of physical and psychological effects after the intervention; however, this timeframe does not allow conclusions regarding the long-term sustainability of behavior and psychological changes.

The groups will receive the designated intervention according to the following allocations: HBG, FxF, or CG. The 2 interventions will be compared to estimate the magnitude of differences in symptom relief and other outcomes, rather than to formally test equivalence. Participants allocated to the CG will be instructed to maintain their usual lifestyle and PA habits throughout the 7-week intervention period and the 4-week follow-up, and not to start any new structured exercise program (eg, supervised gym training, personal training, online exercise programs, or new exercise routines performed ≥2 times/wk). CG participants will receive no supervised training sessions or exercise prescription during the study period. To monitor potential contamination, all participants (including CG) will be asked at baseline, postintervention, and follow-up whether they started, stopped, or substantially changed their PA routines during the study. In addition, CG participants will be contacted weekly by message or phone call to confirm that no new structured exercise program was initiated and to record any relevant changes in PA behavior. Any initiation of a new structured exercise program during the trial will be documented and reported. These participants will remain in the intention-to-treat (ITT) analysis, and a per-protocol sensitivity analysis excluding participants with major protocol deviations will be conducted.

In addition to exercising, participants will receive educational instruction about their disease, surgery, eating habits, and PAs. A summary of the frequency, intensity, type, time, volume, and progression (FITT-VP) principles, as well as the progression factors proposed for each program modality can be found in Table 2. Researchers who participate in the clinical trial will undergo specific training to standardize the intervention. The professionals have more than 10 years of experience in the field of physical exercise and prehabilitation for individuals with obesity.

**Table 2.** Principles for each exercise modality group.

Principles	Home-based group	Face-to-face group	Control group
Frequency	2 d/wk	2 d/wk	Maintain usual physical activity habits (no new structured exercise program)
Intensity	Moderate	Moderate	Maintain usual physical activity habits (no new structured exercise program)
Time or volume	Aerobic: 30 min of aerobic training on a treadmill, stationary bike, or walking outdoors Resistance training: 7 exercises targeting the major muscle groups 3 sets of 8-10 repetitions for each exercise Approximately 2 s for each concentric contraction and 4 s for eccentric phases 40‐60 s between sets ~40 min per section	Aerobic: 30 min of aerobic training on a treadmill Resistance training: 7 exercises targeting the major muscle groups 3 sets of 8-10 repetitions for each exercise Approximately 2 s for each concentric contraction and 4 s for eccentric phases 40‐60 s between sets ~40 min per section	Maintain usual physical activity habits (no new structured exercise program)
Progression factors^a^	Aerobic: Speed Slope of the surface Resistance training: Load (body weight vs external load) Coordination element The number of exercises, sets, and repetitions	Aerobic: Speed Slope of the surface Resistance training: Load (external load; machines) Coordination element The number of exercises, sets, and repetitions	Maintain usual physical activity habits (no new structured exercise program)

a Progression: Aerobic training will involve 5%-10% increases in speed or incline every 2 wk (target: 60%-70% Heart Rate Reserve (HRR); Borg CR-10 scale=4-6). Resistance training will involve ~5% load increases after achieving 10 repetitions, maintaining 3×8-10 repetitions with 40-60 s of rest.

Intervention fidelity will be ensured through standardized training of the professionals involved, structured intervention protocols, and systematic documentation of session delivery. For the HBG, asynchronous video verification will also be used to assess exercise execution and adherence. Participants will be asked to submit video recordings of selected sessions, which will be reviewed by the research team to ensure correct performance and fidelity to the prescribed protocol.

#### Home-Based Program

The protocol will consist of 2 parts: aerobic training and resistance training, and it will last approximately 70 minutes and be performed twice a week on alternate days. The training program was developed specifically for individuals with obesity and can be applied in any environment.

At the beginning of the program, one of the researchers will meet with the participants online (via Google Meet) to explain how the training protocol will be conducted and how the exercises should be performed, and to answer any questions that may arise. Additionally, the meeting will be recorded and made available for participants to access, along with specific videos explaining each exercise. Participants will also have direct contact with the training supervisor for assistance with exercise execution. To monitor the training sessions, participants will be instructed to send a message informing the research team of the completion of the exercises and the time, and the researchers will collect this information weekly. The exercise program will consist of 30 minutes of aerobic training on a treadmill, stationary bike, or walking outdoors, followed by 30 minutes of strength exercises. The Borg scale (CR-10) will be used to monitor exercise intensity, and it is recommended that effort be maintained between 4 and 6, considered a moderate zone of effort, for both aerobic and strength exercises [39], or a 5% increase in speed or incline will be implemented every 2 weeks and in the load of resistance exercises after mastering 10 repetitions (3 sets of 8-10 repetitions, 40-60 seconds of rest). The strength exercises for the upper limbs will include arm flexion (inclined), cable row, and shoulder press, and those for the lower limbs will include free squats, lateral lunges, stepping hip thrusts, and plantar flexions. The exercises can be adapted if the participant has any mobility restrictions or is unable to perform any of the exercises.

#### Face-to-Face Program

Two researchers with experience in interventions for individuals with obesity will conduct the training sessions at the physiotherapy clinic at UDESC. The sessions will be held in the afternoon and last approximately 70 minutes. The first week will be dedicated to familiarizing participants with the exercise protocol.

**Figure 2.**
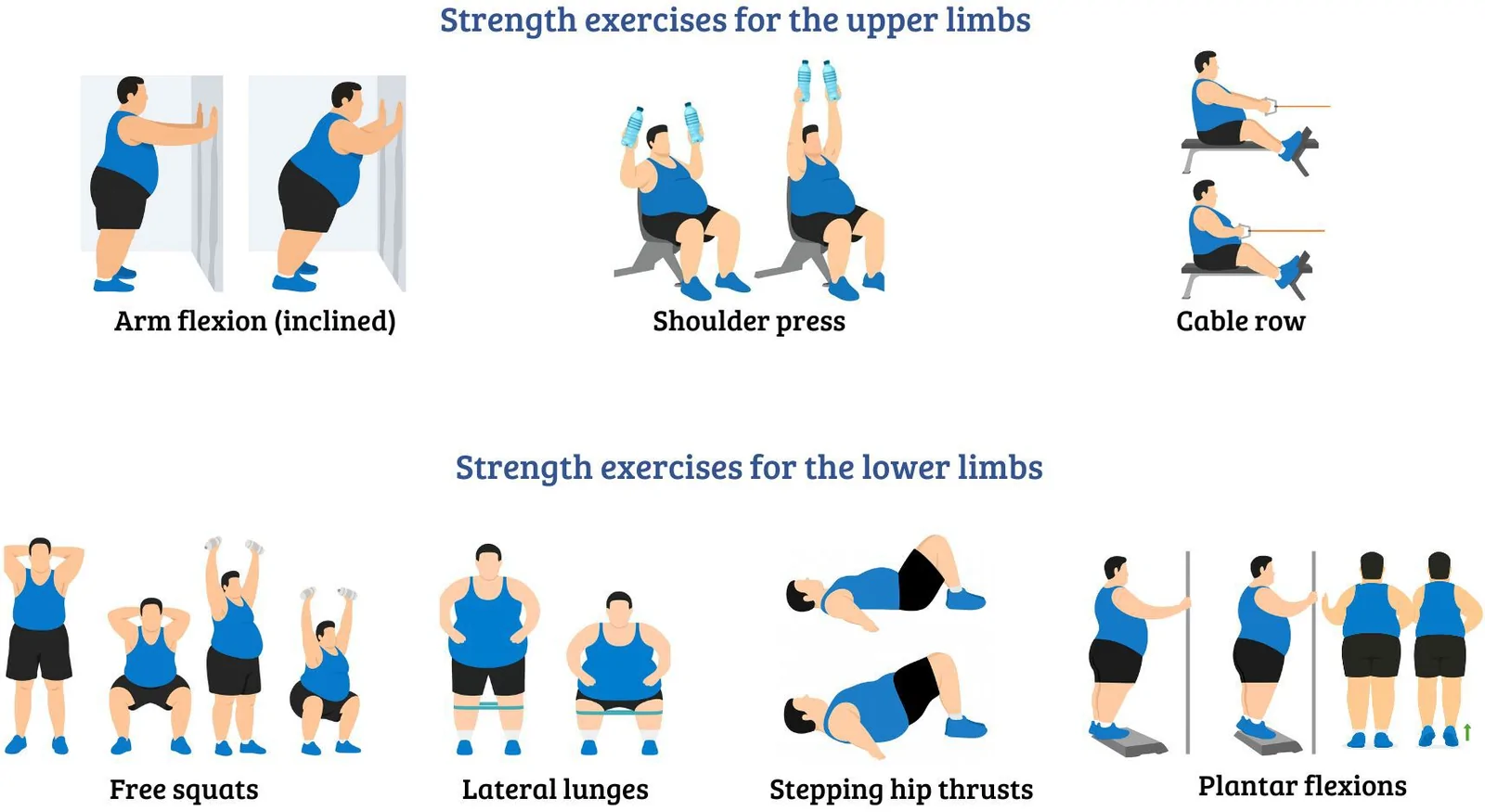
Illustration of resistance exercises.

Initially, participants will perform 30 minutes of aerobic exercise (treadmill or stationary bike). Once this stage is complete, they will begin a protocol of strength exercises, which will include arm flexion (incline), cable row, and shoulder press. The exercises for the lower limbs will be free squats, lateral lunges, stepping hip thrusts, and plantar flexions, and participants will perform the training in the same manner as the HBG. The loads from all training sessions of the FxF group patients will be recorded, with patients being encouraged to reach concentric failure (ie, the inability to perform one more repetition) in the last series, as prescribed. We chose to prescribe training using maximum repetitions, where the number of repetitions to be performed is fixed and load variations occur based on this reference. A progressive increase in the load level will be initiated when the patient correctly performs the exercises with an ideal movement pattern for 2 consecutive days. Figure 2 presents an illustration of the resistance exercises.

#### Health Education

The health education intervention will consist of 3 previously recorded videos, which will be made available after the week of familiarization, between the second and third weeks of the intervention, and between the fourth and fifth weeks of training.

The sessions will be recorded by physiotherapists and will address essential aspects of the relationship between PA and BMS. The videos will each be a maximum of 10 minutes long and will be structured to achieve the following objectives: (1) understand the importance of PA in the context of BMS, (2) identify and overcome barriers to the regular practice of PA, (3) learn behavioral and cognitive strategies to establish a more active lifestyle, and (4) create a personalized action plan, with realistic and sustainable goals.

Each video will address the 3 pillars of the self-determination theory (SDT): autonomy, competence, and relatedness, in addition to the steps called the “5As”: assess, advise, agree, arrange, and assist [40-42]. To ensure intervention fidelity and quality, participant engagement will be monitored via video analytics (defining adherence as ≥80% viewing completion). Furthermore, the content has been culturally adapted to the Brazilian context, featuring Portuguese audio with subtitles for accessibility. To specifically address and mitigate obesity-related stigma, self-compassion strategies have been embedded throughout the video content, reinforcing the “autonomy” pillar.

Table 3 provides a detailed view of the contents of each session, aligning with the pillars of the SDT and the steps of the “5As.”

**Table 3.** Content of health education videos.^a^

Content	Integration with SDT^b^	5As^c^
Health education 1: Get moving: the first step to change^d^		
Introduce the program and integrate participants Explain the importance of PA^e^ in prehabilitation for BMS^f^ Discuss the impacts of a sedentary lifestyle and the benefits of an active lifestyle Reflect on individual perceptions, barriers, and motivations related to PA Establish progressive short-, medium-, and long-term goals Create an individualized plan to support ongoing engagement.	Autonomy: personal choices about where to begin Competence: highlighting benefits and small past victories Relationships: welcoming and integrating into the group	Assess: identify history, routine, barriers, and facilitators Advise: offer guidance on the benefits of PA and risk mitigation
Health education 2: Building habits and overcoming barriers		
Teach participants how to establish an active routine and differentiate it from structured PA Identify positive environmental cues that can increase PA Explore strategies to eliminate indicators of inactivity and encourage active habits Introduce the concept of positive reinforcement to make PA more enjoyable Identify solutions to common obstacles (eg, lack of time, fatigue, low motivation)	Autonomy: the participant chooses their goals and strategies Competence: training skills to maintain an active routine Relationships: support from the team and peers	Agree: co-define realistic goals aligned with the participant’s values Assist: teach self-monitoring, action planning, and coping skills, positive reinforcement, and practical environmental adjustments
Health education 3: Committing to change		
Differentiate between extrinsic and intrinsic rewards for maintaining PA Recognize sources of support and strategies for maintaining long-term motivation Formalize a personal contract to commit to behavior change	Autonomy: commitment contract and adjustments made by the participant Competence: recognizing achievements and consolidating self-efficacy Relationship: mobilizing ongoing social support	Arrange: organize follow-up, review and adjust goals, consolidate support networks, and schedule follow-ups

a The health education intervention will be developed by the authors themselves and offered to both intervention groups, aiming to provide participants with knowledge and practical tools to increase their adherence to the exercise program, thereby promoting a more active lifestyle before undergoing BMS.

b SDT: self-determination theory.

c 5As: assess, advise, agree, arrange, and assist.

d Before the start of the exercise intervention.

e PA: physical activity.

f BMS: bariatric and metabolic surgery.

### Outcome Measures

#### Primary Outcome Measure

**Figure 3.**
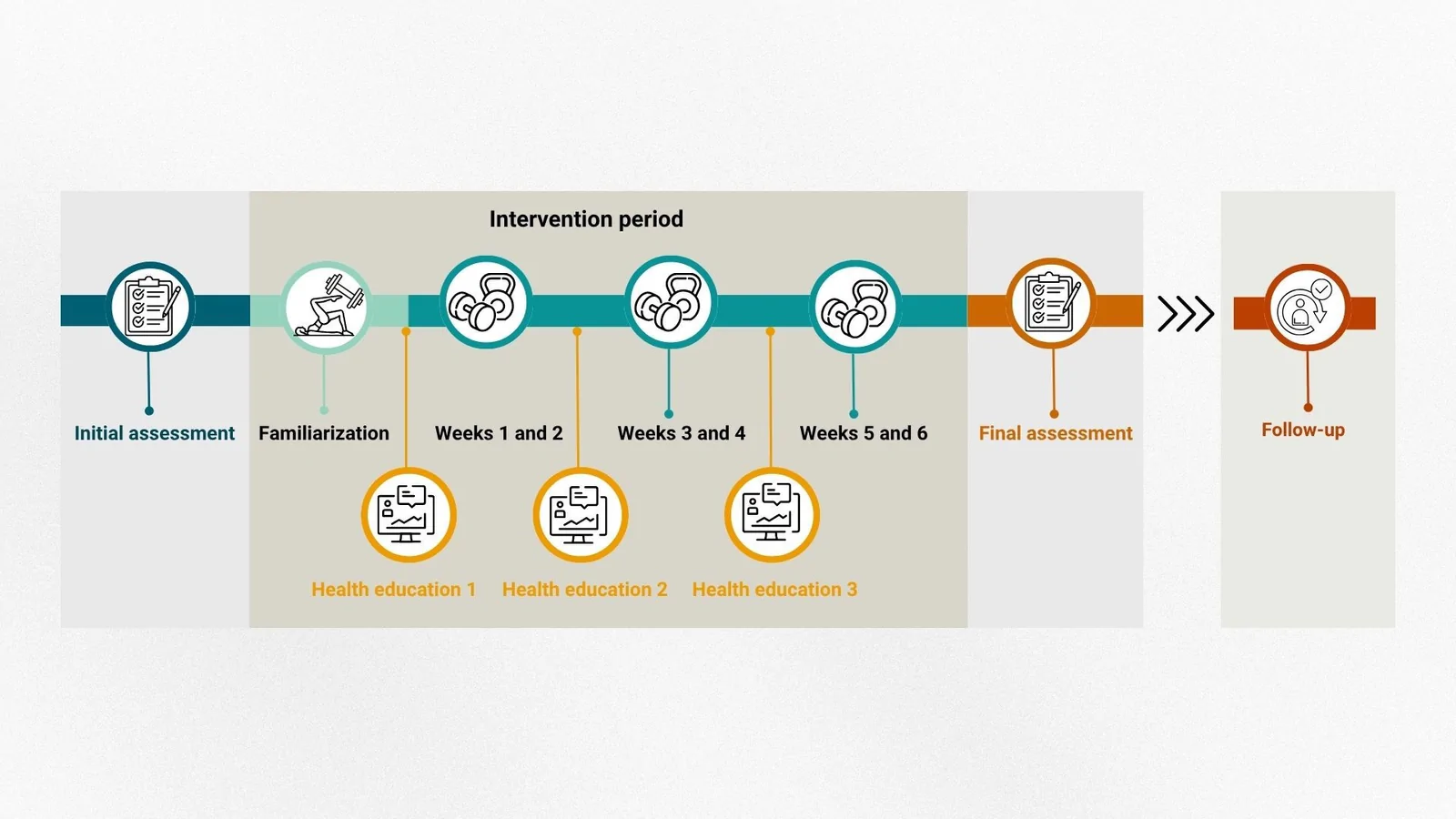
Schematic drawing of the in-person and home-based prehabilitation program protocol.

Assessments will be conducted before the start of the intervention, after the 7-week program, and at the 4-week follow-up (Figure 3). Data collection will be conducted in person at the UDESC facilities for both the face-to-face and home-based groups. In the initial assessment, a characterization questionnaire will be administered to obtain socioeconomic and health information (age, marital status, educational level, occupation, PA level, and main symptoms). Patient data will be maintained solely by the principal researcher to protect confidentiality throughout the study, from inception to conclusion. The variables analyzed, along with their respective instruments, are listed below.

The primary outcome will be the total score of the Hospital Anxiety and Depression Scale (HADS). The HADS was developed in England [43] and was validated for Brazilian Portuguese by Botega et al [44]; the instrument showed internal consistency (Cronbach α) of 0.68 for anxiety and 0.77 for depression in its validation study. The instrument is a scale completed through an interview, preferably answered by the patient, and contains 14 questions, of which 7 assess anxiety (HADS-A) and 7 assess depressive symptoms (HADS-D). The scale emphasizes the psychological signs or consequences of anxiety and depression, excluding clinical symptoms such as dizziness and headache. The questions alternate, with half of them written in a positive tone and the other half in a negative tone. Each question is assigned a score from 0 to 3, with 3 indicating a state associated with more depressive symptoms or anxiety. Analyses of the HADS-A and HADS-D subscales will be conducted as secondary exploratory outcomes. In this study, HADS scores ≥8 will be used as a cutoff for each domain, as this is the score that indicates the presence of depressive and anxious symptoms and possible cases of depression and anxiety.

#### Secondary Outcome Measures

Secondary outcomes will include analyses of the HADS subscales (HADS-A and HADS-D), sleep quality assessed using the Pittsburgh Sleep Quality Index (PSQI), mood states assessed using the Brunel Mood Scale (BRUMS), quality of life assessed using the World Health Organization Quality of Life–BREF (WHOQOL-BREF), and self-efficacy assessed using the Physical Exercise Self-Efficacy Scale. Physical function will be assessed using the 6MWT, handgrip strength, and the 5-repetition sit-to-stand test. Adherence will be evaluated according to group allocation: attendance at supervised sessions for the FxF group, and, for the HBG, aerobic exercise recorded via the Strava smartphone app (Strava, Inc) and resistance training reported via text message.

The PSQI, developed by Buysse et al [45], demonstrates high internal consistency (global Cronbach α=0.83). It consists of 19 questions assessing the quality and pattern of sleep, grouped into 7 components: subjective quality, latency, duration, habitual efficiency, disorders, medication use, and daytime dysfunction. Each component is evaluated on a scale of 0 to 3, yielding a global score (range 0‐21); a score ≥5 indicates poor sleep quality.

Mood states will be evaluated using the BRUMS [46]. Validated for the Brazilian population, the instrument shows robust psychometric properties, with subscale Cronbach α coefficients ranging from 0.70 to 0.85 (eg, tension α=0.78) [47]. It consists of 24 items across 6 domains: tension, depression, anger, vigor, fatigue, and mental confusion. Participants rate each item on a 5-point scale (0=“not at all” to 4=“extremely”), resulting in a total score ranging from 0 to 16 for each mood state.

Preoperative quality of life will be assessed using the WHOQOL-BREF instrument, developed by the World Health Organization [48]. Validated for the Brazilian population, the instrument demonstrates satisfactory internal consistency (Cronbach α=0.91) [49]. This self-assessment questionnaire consists of 26 items: 2 general questions regarding overall quality of life and health, and 24 items distributed across 4 domains: physical health, psychological, social relationships, and environment. The instrument enables a comprehensive, multidimensional assessment, in which higher scores indicate a better perception of quality of life.

To assess self-efficacy, the Physical Exercise Self-Efficacy Scale will be used [50]. This instrument consists of 5 items that assess an individual’s confidence in performing physical exercises under different emotional states. The items are as follows: feeling worried and in trouble, feeling depressed, feeling nervous, feeling tired, and feeling busy. The instrument was translated, culturally adapted, and validated for the Portuguese population by Martins et al [51]. The questionnaire can be self-administered or administered via interview. Each item is graded on a 5-point Likert scale, defined as follows: 1=“not true at all,” 2=“hardly true,” 3=“probably true,” and 4=“exactly true.”

The interviewer should take care not to influence the patient’s response. When a respondent does not understand the meaning of a question, the interviewer should reread the question slowly, without using synonyms, and neither the question nor its meaning should be discussed, nor should the answer scale be explained. In cases where this is impossible (illiteracy, severe visual impairment, or lack of clinical condition), the instrument is administered by the interviewer, and the effort to avoid influencing the individual’s answers should be redoubled. The score for each facet is the corresponding value marked, and the average of the items is used to obtain the score for each domain answered. The final score is calculated by averaging the scores of all domains (physical, psychological, social relationships, and environment) and ranges from 0 to 100 points. The higher the score, the better the quality of life in the domain or overall.

The 6MWT will be used to assess functional capacity, following the recommendations of the American Thoracic Society [52]. The procedure will be conducted by a trained evaluator in a standardized corridor at the university. Participants will be instructed to walk the longest distance possible for 6 minutes, receiving standardized encouragement. Heart rate, SpO₂, blood pressure, and subjective perception of exertion (perceived exertion using the Borg scale for dyspnea and lower limb fatigue) will be monitored at baseline, every 2 minutes, and at the end of the test. Continuous monitoring of heart rate and SpO₂ will be performed using a pulse oximeter. For greater reliability, each participant will perform 2 tests on the same day, with a minimum interval of 20 minutes between them. The longest distance covered will be considered for analysis.

Handgrip strength will be assessed using a Saehan DHD-1 digital dynamometer, following the recommendations of the American Society of Hand Therapists [53]. Participants will be seated comfortably in an armless chair with their feet flat on the floor, hips and knees flexed at approximately 90°, shoulders adducted and neutrally rotated, elbows flexed at 90°, forearms in a neutral position, and wrists in a range of 0° to 30° extension and 0° to 15° adduction. Three consecutive 3-second maximal contractions will be performed with each hand, with intervals of 30 seconds between attempts and 2 minutes between hands. The arithmetic mean of the 3 tests for each limb will be used for analysis.

The 5-repetition sit-to-stand test will be used to assess lower limb strength, balance control, fall risk, and functional capacity [54,55]. The participant will remain seated in a standardized chair, with feet flat on the floor and arms crossed across the chest, and will be instructed to stand up and sit down 5 consecutive times as quickly as possible. Before and after the test, heart rate, SpO₂, and perception of dyspnea will be recorded using the modified Borg scale. The total time to complete the test will be considered for analysis.

Adherence will be assessed based on the characteristics of each group. In the in-person group, attendance at supervised training sessions will be considered. In the HBG, adherence to aerobic exercise will be monitored by recording activities on the Strava app, while resistance training will be tracked using the Physitrack app (Physitrack PLC), which provides video and text instructions, automatic reminders, and self-monitoring functions. This tool has already demonstrated good acceptability in home-based programs and contributed to greater participant engagement [56]. Based on the records obtained, it will be possible to quantify the proportion of completed sessions relative to the total number planned, as well as verify training feedback and access to the health education videos. For analysis purposes, good adherence will be considered to be participation in at least 75% of the proposed sessions, a value close to that adopted in international multimodal prehabilitation programs that used more rigorous cutoffs, such as 80% [57].

Different approaches to measuring adherence across groups are justified by the distinct modes of intervention delivery. Adherence will be reported descriptively and explored in sensitivity analyses; no formal moderation analyses based on adherence are planned.

### Adverse Events

Any adverse effects observed or reported by patients will be documented and taken into consideration in the study’s results. In addition, these patients will be referred for medical care for proper treatment.

### Statistical Methodology

#### Sample Size Calculation

The G*Power 3.1 (Heinrich-Heine-Universität Düsseldorf) program was used to determine the sample size. The calculation was based on the HADS total score, which is defined as the primary outcome of the study, assuming a repeated-measures design with 3 groups (home-based, face-to-face, and control) and 3 assessment time points (baseline, postintervention, and 4-week follow-up). Considering an α level of .05 and power of .80, an effect size of *f* of 0.25 (moderate) was assumed for the group × time interaction, with an assumed correlation among repeated measures of 0.50 and a nonsphericity correction (ε) of 1.0. Under these assumptions, a sample of at least 42 participants, randomized into the 3 groups, is required. This sample size was calculated to detect differences between groups and was not designed to formally demonstrate equivalence between the interventions. Anticipating a dropout rate of approximately 30% (18/60), a total of 60 participants will be recruited to ensure the necessary final sample size of 42 participants.

#### Statistical Analysis

The analysis will be conducted using the ITT principle, including all randomized participants. Per-protocol sensitivity analyses will also be performed. For continuous variables, a linear mixed model will be used, with fixed effects for group (face-to-face, home-based, and control), time (baseline, postintervention, and follow-up), and group × time interactions. Participants will be modeled as a random effect. Baseline values will be included as part of the repeated-measures structure rather than as covariates, allowing the estimation of within- and between-group changes over time. Age and sex will be considered as covariates in adjusted models if baseline imbalances or clinically relevant associations with the outcomes are identified. The home-based vs face-to-face comparison will be interpreted based on effect estimates and confidence intervals and will not be treated as a formal equivalence analysis. This model, which treats repeated within-participant measures as a random effect, will be used. An appropriate covariance structure for the repeated measures will be selected based on model fit criteria. This method allows for the management of missing data under the assumption that they are missing at random. In cases of systematic losses, multiple imputations may be used as a complementary analysis.

For categorical or dichotomous variables, the chi-square or Fisher exact tests will be applied, where appropriate. For all hypothesis tests, the α level for statistical significance will be set at *P*<.0, corresponding to a 95% confidence level. To assess the magnitude of significant differences between the preintervention and postintervention moments and between groups, the Hedge *g* (x ®1–x ®2⁄ SD grouped) will be calculated. The effect size magnitudes will be interpreted as follows: <0.2, no effect; 0.2 to 0.4, small; 0.5 to 0.7, moderate; and ≥0.8, large [58]. Secondary outcomes will be analyzed using the same modeling strategy; however, these analyses will be considered exploratory, and no formal adjustment for multiple comparisons is planned. The data will be analyzed using IBM SPSS software, version 20.0, licensed to UDESC, and Microsoft Office Excel for Windows.

## Results

In August 2025, this clinical trial received funding from the Research Support Program of the UDESC. Participant recruitment and data collection are scheduled to begin in April 2026. Initial assessments and randomization are expected to occur between April and May 2026. The intervention phase is scheduled to be completed in September 2026, followed by data analysis between October and November 2026. Results are expected to be published in mid-2027.

## Discussion

This protocol for a randomized controlled trial (RCT) aims to evaluate the impact of face-to-face and home-based rehabilitation models, compared with a CG without a structured intervention, in the preparation of individuals with severe obesity who are candidates for BMS. Both models combine physical exercise and health education, focusing on improving functional capacity, exercise self-efficacy, quality of life, mental health, and sleep. The primary hypothesis is that the interventions will produce effects superior to those of the control and that the home-based model will exhibit greater adherence by eliminating logistical barriers, such as travel to the intervention site.

It is well established that prehabilitation has been established as a promising approach to optimize functional status and reduce perioperative risks [59,60]. In the context of obesity, studies have reported benefits including reduced BMI, improved physical fitness, enhanced respiratory muscle strength, and positive effects on metabolic parameters [61-65]. However, the literature is currently limited by high methodological heterogeneity regarding exercise type, duration, and intensity, which hinders standardization and limits comparability between studies [66,67].

Previous studies have demonstrated diversity in preoperative interventions for patients undergoing bariatric surgery, while also highlighting essential gaps in care. García-Delgado et al [15] tested respiratory training and measured outcomes related to preoperative weight, while the Bari-Active RCT [35] used a 6-week behavioral intervention with objective monitoring of PA, demonstrating benefits for quality of life. A recent review of home-based prehabilitation [68] indicated that home-based programs can reduce complications and improve functional performance, as well as symptoms of depression and anxiety. However, direct comparisons between home-based and in-person protocols, integrating objective measures of PA and mental health outcomes, are still scarce; this study addresses this gap by standardizing a multimodal intervention using FITT-VP principles, positioning it to resolve current inconsistencies in the evidence.

The face-to-face model enables close monitoring by the multidisciplinary team, fostering the therapeutic bond and facilitating direct observation of the patient’s performance—factors associated with improved clinical outcomes [69,70]. On the other hand, this model presents potential barriers, such as lack of time, travel costs, and access difficulties, especially for individuals in remote areas or with lower socioeconomic status [71].

The home-based model, supported by digital technologies, emerges as a viable alternative, expanding the reach of rehabilitation and maintaining remote professional support through video calls, online platforms, and health apps [72]. Studies show that, when well-structured, home-based programs can achieve functional and clinical outcomes similar to those of face-to-face programs, while also favoring adherence and reducing hospital costs [68,73,74]. In addition, home-based rehabilitation can reduce postoperative complications, length of hospital stay, and psychological symptoms, such as anxiety and depression, while maintaining high levels of participation [68,75-78].

The strengths of this study include rigorous adherence to the SPIRIT guidelines and the detailed description of the intervention protocol using FITT-VP principles, ensuring reproducibility and allowing for direct comparisons between groups. The multimodal intervention, which integrates exercise and health education, can facilitate significant changes in physical, psychological, and behavioral aspects, all of which are fundamental for improving obesity-related outcomes.

Crucially, the study prioritizes high ecological validity and generalizability through a stratified recruitment strategy (targeting 50% participants from low-socioeconomic backgrounds and 20% from rural populations). This approach actively mitigates the selection bias often found in university-based trials and ensures the results reflect diverse, real-world clinical conditions, supporting future broad implementation in public health systems.

The innovation of this study lies in the methodological evaluation of 2 structured prehabilitation formats in the context of BMS, considering physical and psychological outcomes as well as adherence. The inclusion of a CG will allow us to isolate the specific effects of the interventions and provide higher-level evidence to support future guidelines.

However, some limitations must be recognized. Psychological outcomes will be assessed using self-reported instruments, which may introduce reporting bias; the follow-up period will be relatively short, limiting conclusions regarding long-term effects. Regarding the HBG, adherence and exercise execution depend on participant engagement outside a supervised environment. To mitigate this, asynchronous video verification strategies will be used (targeting ≥85% of execution fidelity) to cross-reference self-reported data. In addition, participant blinding will not be feasible due to the nature of the interventions, although outcome assessors and statisticians will remain blinded. This lack of blinding may introduce expectation effects that could influence self-reported psychological outcomes.

Nevertheless, if the hypotheses are confirmed, the results may guide the implementation of prehabilitation programs adapted to the needs and realities of the participants, favoring individual-centered preoperative care and contributing to health policies at lower costs. In addition, they will provide support for the incorporation of evidence-based strategies in face-to-face and home-based modalities, offering safe and affordable options to optimize surgical preparation and improve perioperative outcomes in people with severe obesity awaiting BMS.

## Supplementary material

10.2196/87053 Checklist 1 SPIRIT checklist.

## Abbreviations

6MWT: 6-minute walk test
BMS: bariatric and metabolic surgery
BRUMS: Brunel Mood Scale
CG: control group
COPD: chronic obstructive pulmonary disease
FITT-VP: frequency, intensity, type, time, volume, and progression
FxF: face-to-face exercise group
HADS: Hospital Anxiety and Depression Scale
HBG: home-based exercise group
ITT: intention-to-treat
PSQI: Pittsburgh Sleep Quality Index
RCT: randomized controlled trial
ReBEC: Brazilian Clinical Trials Registry
SDT: self-determination theory
SPIRIT: Standard Protocol Items: Recommendations for Interventional Trials
SpO₂: peripheral oxygen saturation
UDESC: Santa Catarina State University
WHOQOL-BREF: World Health Organization Quality of Life–BREF
